# Detection of *Aeromonas hydrophila* by Basic and Fluorescent MIRA Assays

**DOI:** 10.3390/microorganisms13092191

**Published:** 2025-09-19

**Authors:** Qiuya Huang, Fa Dai, Lujia Su, Miaomiao Zhang, Xinjie Miao, Yujie Ding, Cheng Xu, Jiehao Xu

**Affiliations:** 1 College of Biological and Environmental Science, Zhejiang Wanli University, Ningbo 315100, China; 2023881095@zwu.edu.cn (Q.H.); 2024881042@zwu.edu.cn (L.S.); 2021015042@zwu.edu.cn (M.Z.); 2022014010@zwu.edu.cn (X.M.); dingyujie0722@163.com (Y.D.); 2 Institute of Animal Sex and Development, Zhejiang Wanli University, Ningbo 315100, China; daifa@zwu.edu.cn; 3 Department of Paraclinical Sciences, Faculty of Veterinary Medicine, Norwegian University of Life Sciences, 1433 Ås, Norway

**Keywords:** *A. hydrophila*, multi-enzyme isothermal rapid amplification (MIRA), sensitive, specific

## Abstract

*Aeromonas hydrophila* is a prevalent opportunistic pathogen in aquaculture. To establish a rapid, convenient, and accurate detection method for *A. hydrophila*, this study developed and evaluated Multi-Enzyme Isothermal Rapid Amplification (MIRA) assays, which could complete amplification within 20 min at a constant temperature of 39 °C. The basic MIRA assay targeting the aerolysin (*aerA*) gene demonstrated high specificity, showing no cross-reactivity with six related bacterial species including *Aeromonas veronii*, *Vibrio harveyi*, *Pseudomonas fluorescens*, *Bacillus subtilis*, *Bacillus cereus*, and *Lactiplantibacillus plantarum*. The fluorescent MIRA assay achieved a detection limit of 1 fg/μL (3.1 × 10^2^ copies/μL) when using the pUC57-aerA standard plasmid, while real-time quantitative PCR achieved a detection limit of 0.1 fg/μL (31 copies/μL). Thus, the MIRA assay exhibited 10-fold lower sensitivity than qPCR but shortened the reaction time from several hours (nearly two hours) to within one hour. Both the specificity and sensitivity of the MIRA reactions were evaluated with three independent experiments. These findings suggested that the developed MIRA assays provide a rapid, specific, and practical diagnostic tool for *A. hydrophila* detection in aquaculture environments, particularly suitable for resource-limited field applications.

## 1. Introduction

The aquaculture industry plays a vital role in the global food supply chain, providing essential biological resources from both marine and freshwater ecosystems [1]. Farmed aquatic species currently account for up to 50% of human consumption [2]. Since the 1960s, the global per capita fish consumption has increased from 9 kg in 1961 to 20.7 kg in 2022 [3,4]. However, the intensification of aquaculture practices has elevated disease risks, with high-density farming contributing to frequent outbreaks that cause substantial economic losses [5]. Infectious diseases now represent a major constraint to sustainable aquaculture development [6]. In particular, since its initial outbreak in 2009, *A. hydrophila* has caused tens of millions of dollars in economic losses to aquaculture [7], underscoring the urgent need for rapid pathogen detection and early warning systems.

*A. hydrophila*, a Gram-negative opportunistic pathogen prevalent in freshwater environments, exhibits strain-dependent pathogenicity correlated with specific virulence factors [8,9,10]. Key virulence determinants include aerolysin (*aerA*), hemolysin (*hlyA*), serine protease, metalloprotease, and cytotoxic enterotoxins (*act*) [11,12,13]. Pathogenic strains caused diverse clinical manifestations across aquatic species, such as motile aeromonad septicemia in fish [14], ascites disease in crabs [15], and septicemia in soft-shelled turtles [16]. Infection severity correlates with extracellular virulence factors [17], necessitating precise detection of virulent strains for effective disease management.

In aquaculture pathogen diagnosis, multiple methodologies have been employed. The most fundamental and conventional approach involves microscopic examination or dissection of the infected organism. However, this approach is highly dependent on human interpretation, introducing a significant degree of subjectivity that may affect the accuracy and reliability of the diagnostic outcomes [18]. In recent years, advances in molecular biology technology have led to the application of nucleic acid molecular diagnostic techniques for detecting various pathogens in aquatic animals. For instance, Polymerase Chain Reaction (PCR) technology identifies pathogens by amplifying specific nucleic acid sequences of microbial agents [19]. Its derivative technologies, including reverse transcription PCR, nested PCR, and real-time fluorescent quantitative PCR, have also been extensively utilized in pathogen detection [20]. While these nucleic acid-based techniques improve specificity, their reliance on thermal cyclers and prolonged processing times (hours) hinders field applications [21,22]. Isothermal amplification technologies such as loop-mediated isothermal amplification (LAMP) address some limitations but face challenges including false negatives, amplification inhibition, and complex result interpretation [23,24,25,26].

Multienzyme isothermal rapid amplification (MIRA) represents an innovative alternative, combining helicase and recombinase enzymes to enable rapid target amplification within 20 min at 37–42 °C. Unlike conventional recombinase polymerase amplification (RPA) systems, MIRA’s dual-enzyme mechanism accelerates reaction kinetics while enhancing tolerance to inhibitors [27]. The process involves recombinase-primer complexes that identify complementary sequences, facilitated by auxiliary proteins to form D-loop structures for polymerase-mediated chain extension at constant temperatures [28]. Molecular detection techniques such as real-time PCR are widely utilized for nucleic acid amplification. However, they require complex instrumentation and specialized expertise for data interpretation, limiting their accessibility [29]. In contrast, MIRA offers superior efficiency and overcomes these constraints. Compared to other isothermal amplification methods, such as loop-mediated isothermal amplification (LAMP), MIRA exhibits a faster reaction rate and enhanced tolerance to potential inhibitors due to its multi-enzyme system [25,26]. Additionally, MIRA is characterized by greater time efficiency and cost-effectiveness relative to alternative amplification techniques [30]. Importantly, this method does not require sophisticated laboratory equipment or highly trained personnel, making it a viable diagnostic tool for deployment in resource-constrained regions [31]. Given these advantages, MIRA holds significant potential for broad application in nucleic acid detection, particularly in settings with limited laboratory infrastructure.

**Figure 1:**
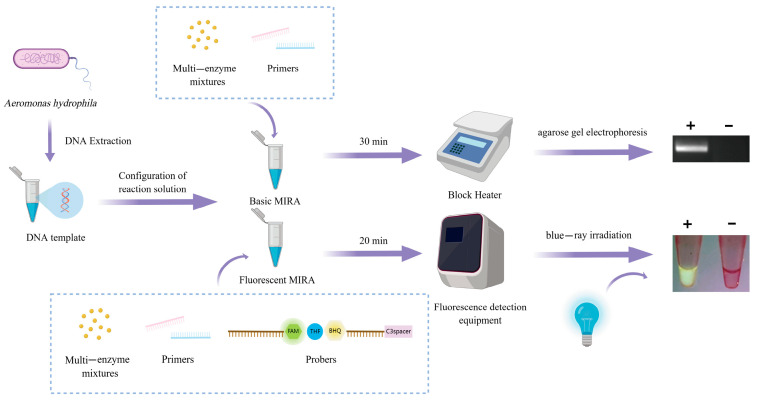
Schematic representation of two MIRA methods for the detection of *A. hydrophila*.

This study presents a novel *aerA* virulence gene detection method leveraging MIRA technology. Characterized by cost-effectiveness, high efficiency, simplicity, and sensitivity, it enables the rapid identification of pathogenic *A. hydrophila*. By combining specificity for virulence determinants with isothermal simplicity, the proposed method addresses critical needs for field-deployable diagnostics in aquaculture disease surveillance, enabling timely intervention against emerging infections. Figure 1 illustrates the detection processes of *A. hydrophila* using two MIRA methods.

## 2. Materials and Methods

### 2.1. Bacterial Strains and Genomic DNA Extraction

The strains *A. hydrophila* (NB-2024-AH-01), *P. fluorescens* (NB-2024-PF-01), *B. subtilis* (NB-2024-BS-01), *B. cereus* (NB-2024-BC-01), and *L. plantarum* (NB-2024-LP-01) were obtained from laboratory collections at Zhejiang Wanli University. *A. veronii* (HZ-2022-AV-06) and *V. harveyi* (HZ-2022-VH-08) were provided by the Institute of Hydrobiology, Zhejiang Academy of Agricultural Sciences.

*A. hydrophila* (NB-2024-AH-01) was cultured in Luria–Bertani (LB) medium at 28 °C; *P. fluorescens* (NB-2024-PF-01) was cultivated in LB medium at 25 °C; *B. subtilis* (NB-2024-BS-01) was grown in LB medium at 37 °C; *B. cereus* (NB-2024-BC-01) was incubated in LB medium at 30 °C; *L. plantarum* (NB-2024-LP-01) was cultured in LB medium at 37 °C; *A. veronii* (HZ-2022-AV-06) was cultured in LB medium at 30 °C; and *V. harveyi* (HZ-2022-VH-08) was cultivated in LB medium at 28 °C.

### 2.2. MIRA Probe and Primers

Primer length of 30–35 bp and amplicon lengths not exceeding 500 bp are recommended for the MIRA reaction. Herein, specificity analysis of the three designed primers was conducted using the Primer Primer5 tool. The *aerA* gene (GenBank Accession No. EU650663.1) was downloaded from the NCBI GenBank database. Primer pairs AH-aerA-F1/R1, AH-aerA-F2/R2, AH-aerA-F3/R3 were used for basic MIRA test and fluorescent MIRA test, and AH-aerA-F/R were used for qPCR.

The probe sequence had four modification sites: a dSpacer (THF) at the middle of the 5′ end as a nucleic acid exonuclease recognition site, a fluorescent group (FAM) upstream, a quenched group (BHQ) downstream, and a C3-Spacer at the 3′ end.

All primers and probes were synthesized at Sangon Biotech Co., Ltd. (Shanghai, China), and the sequence of all the primers and probe are summarized in Table 1.

### 2.3. Construction of Recombinant Plasmid

The *aerA* gene was chemically synthesized by Sangon Biotech Co., Ltd. (Shanghai, China). Then, the synthesized *aerA* gene was cloned into the pUC57 vector using T4 DNA ligase. Afterward, the recombinant vector pUC57-aerA was transformed into *Escherichia coli* Top10 competent cells for subsequent experiments [32]. Top10/pUC57-aerA was cultured in LB medium supplemented with ampicillin (100 μg/mL) at 37 °C.

### 2.4. Nucleic Acid Extraction

The genomic DNA of bacteria was extracted using a DNA extraction kit (Tiangen Bio-Tech, Beijing, China). The plasmid of Top10/pUC57-aerA was extracted using a Mini Plasmid Kit (Tiangen Bio-Tech, Beijing, China). All DNA extracts had a concentration ranging from 100 to 500 ng/μL and an A_260_/A_280_ ratio between 1.8 and 2.0. All these data were measured using a NanoDrop One spectrophotometer (Thermo Fisher Scientific, Waltham, MA, USA). All DNA templates were stored at −20 °C until the assays were performed.

### 2.5. Basic Multienzyme Isothermal Rapid Amplification

#### 2.5.1. Primer Screening for the Basic MIRA Assay

Three pairs of primers were designed specifically for the *aerA* gene fragment of *A. hydrophila*, namely AH-aerA-F1/R1, F2/R2, and F3/R3 (Table 1). MIRA reactions were performed using a DNA multienzyme isothermal rapid amplification basic kit (AMP-Future Biotech Co., Ltd., Changzhou, China). The mixture was prepared in a tube containing buffer A (29.4 μL), 10 μM of upstream primer (2 μL), 10 μM of downstream primer (2 μL), 11.5 μL of ddH_2_O, and 2 μL of extracted genomic DNA template. Next, 2.5 μL buffer B was added to the tube, then the tube was shaken up and down to mix, and the reaction tube was placed in a constant temperature metal bath at 39 °C for 30 min. Agarose gel electrophoresis was conducted using a gel prepared with 2% (*w*/*v*) agarose (Sangon Biotech Co., Ltd., A620014-0100) dissolved in 1× TAE buffer (Sangon Biotech Co., Ltd., B548101-0500). The same 1× TAE buffer was employed as the running buffer, and the electrophoresis was carried out at a constant voltage of 140 V.

Following the reaction, proteins in the reaction products were denatured by an extraction method. To each reaction tube, 50 μL of Tris-saturated phenol (Beijing Coolaber Technology Co., Ltd., Beijing, China, SL2020)/chloroform (Sinopharm Chemical Reagent Co., Ltd., Shanghai, China, 10006818)/isoamyl alcohol (Sinopharm Chemical Reagent Co., Ltd., 10003218) (25:24:1) DNA extraction solution was added. The solution was mixed and then centrifuged at 12,000 rpm (Eppendorf 5424R, FA-45-24-11 fixed—angle rotor) for 5 min at 4 °C. Subsequently, 5 μL of the supernatant was mixed with 1 μL of 6 × loading buffer (Sangon Biotech Co., Ltd., B548315-0001) for agarose gel electrophoresis. The purpose of this step is to eliminate proteins from the nucleic acid amplification products.

#### 2.5.2. Specificity of the Basic MIRA Assay

The specificity of the MIRA reactions was evaluated using six bacterial strains, including *A. veronii*, *V. harveyi*, *P. fluorescens*, *B. subtilis*, *B. cereus*, and *L. plantarum*. Genomic DNA was extracted and MIRA amplification reaction was performed with primers AH-aerA-F2/R2 using the protocol in Section 2.5.1. Plasmid pUC57-aerA and *A. hydrophila* were used as positive controls, and distilled water was used as negative control. The MIRA reaction solution was denatured and analyzed by agarose gel electrophoresis. The specificity of the MIRA reactions was evaluated with three independent experiments.

#### 2.5.3. Sensitivity of the Basic MIRA Assay

The recombinant plasmids pUC57-aerA was 10-fold serially diluted to obtain seven different concentrations of the DNA template (10 pg/μL, 1 pg/μL, 100 fg/μL, 10 fg/μL, 1 fg/μL, 0.1 fg/μL, and 0.01 fg/μL) to determine the detection limit of the MIRA assay. MIRA reaction was performed with primers AH-aerA-F2/R2 using the protocol in Section 2.5.1. The distilled water was used as negative control, and the sensitivity of the MIRA reactions were evaluated with three independent experiments.

### 2.6. Fluorescent Multienzyme Isothermal Rapid Amplification

#### 2.6.1. Primer Screening for the Fluorescent MIRA Assay

The three upstream primers and three downstream primers were used in different combinations with probe to perform fluorescent MIRA using *A. hydrophila* genomic DNA as template. MIRA reaction was performed using a DNA multienzyme isothermal rapid amplification exo kit (AMP-Future Biotech Co., Ltd.). The mixture was prepared in a tube containing buffer A (17.4 μL), 10 μM of upstream primer (1 μL), 10 μM of downstream primer (1 μL), 10 μM of probe (0.3 μL), 4.25 μL of ddH_2_O, and 2.5 μL of extracted genomic DNA template. Next, 1.25 μL buffer B was added to the tube, and then the tube was shaken up and down to mix. MIRA reaction was performed on real-time fluorescence quantitative PCR (Roche Light Cycler 480, Basel, Switzerland) with a fluorescence reaction program at 39 °C. Fluorescence signals were collected every 30 s, and the reaction time was 20 min.

#### 2.6.2. Specificity of the Fluorescent MIRA Assay

The specificity of the established fluorescence MIRA method was evaluated using the extracted genomic DNA of six bacterial strains, including *A. veronii*, *V. harveyi*, *P. fluorescens*, *B. subtilis*, *B. cereus*, and *L. plantarum*. The plasmid pUC57-aerA was used as a positive control, and distilled water was used as the negative control. MIRA reaction was performed using the protocol in Section 2.6.1.

#### 2.6.3. Sensitivity of the Fluorescent MIRA Assay

The plasmid pUC57-aerA was 10-fold serially diluted to obtain seven different concentrations of the DNA template to determine the detection limit of the MIRA reaction. MIRA reaction was performed using the protocol in Section 2.6.1. To compare the detection limit of MIRA with qPCR method, seven concentrations of DNA template were subjected to qPCR reactions using the primer pair AH-aerA-F/AH-aerA-R and ChamQ Universal SYBR qPCR Master Mix (Vazyme Biotech Co., Ltd., Nanjing, China). The reaction system was as follows: 5 μL of 2 × ChamQ Universal SYBR qPCR Master Mix, 10 μM of upstream primer (0.2 μL), 10 μM of downstream primer (0.2 μL), 3.6 μL of ddH_2_O, and 1 μL of template. The qPCR reaction procedure was as follows: 95 °C for 30 s, followed by 40 cycles of 95 °C for 10 s and 60 °C for 30 s. The melt curve program was set at 95 °C for 15 s, 60 °C for 60 s, followed by 95 °C for 15 s. The sensitivity of the qPCR reactions was evaluated with three independent experiments.

## 3. Results

### 3.1. Basic MIRA Assay

#### 3.1.1. Optimal Primer Set for the Basic MIRA Assay

**Figure 2:**
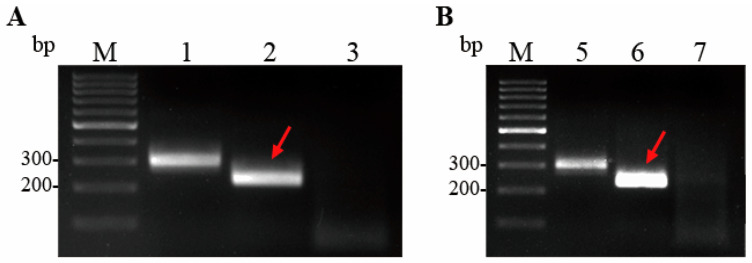
Screening of MIRA primer pairs. (**A**) The amplification results of basic MIRA using the pUC57-aerA plasmid as template. M: 1000 bp DNA Marker; Lane 1: primer pair AH-aerA-F1/R1; Lane 2: using primer pair AH-aerA-F2/R2; Lane 3: primer pair AH-aerA-F3/R2. The lane indicated by the red arrow represents the primer pair finally selected for the basic MIRA detection method. (**B**) The amplification results of basic MIRA using primer pair AH-aerA-F2/R2. M: 1000 bp DNA Marker; Lane 5: the positive control template and positive control primers from the kit were used. The “positive control template” and “positive control primers” refer to a plasmid sequence developed by Anpu Future Biotechnology Co., Ltd. (Changzhou, China). This plasmid sequence can be stably amplified through the basic MIRA technology; Lane 6: using the genomic DNA of *A. hydrophila* as temple; Lane 7: negative control using nuclease-free water. The lane indicated by the red arrow contains the genomic DNA of *A. hydrophila* from the sample to be tested.

Based on the *aerA* gene sequence from *A. hydrophila*, three pairs of primers were designed and screened using the pUC57-aerA plasmid as a template for the basic MIRA. As shown in Figure 2A, the MIRA amplification products derived from the three pairs of primers using the pUC57-aerA plasmid as a template all showed distinct single band in the agarose gel electrophoresis, and no amplification was observed in the negative control, suggesting that all three pairs of primers can specifically target and amplify the *aerA* gene of *A. hydrophila*. The amplification band generated by the primers AH-aerA-F2/R2 was the brightest, with a size between 200 bp and 300 bp. Therefore, the primer pair AH-aerA-F2/R2 was chosen.

As shown in Figure 2B, the band intensity of MIRA detection of the genomic DNA of *A. hydrophila* was comparable to that using the pUC57-aerA plasmid as a template, and there was no non-specific amplification. This indicates that the MIRA detection method using primer pair AH-aerA-F2/R2 was highly effective for detecting the genomic DNA of *A. hydrophila*.

#### 3.1.2. The Basic MIRA Assay with High Specificity

**Figure 3:**
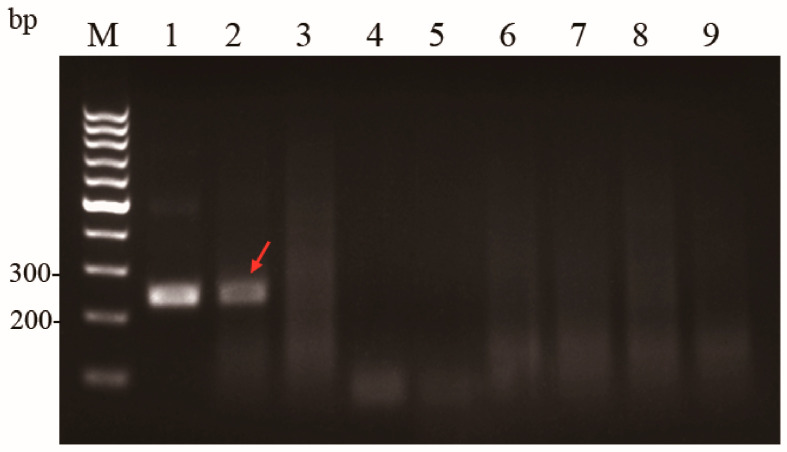
Specificity of the basic MIRA assay. M: 1000 bp DNA Marker; Lane 1: pUC57-aerA; Lane 2: *A. hydrophila*; Lane 3: *A. veronii*; Lane 4: *V. harvey*; Lane 5: *P. fluorescens*; Lane 6: *B. subtilis*; Lane 7: *B. cereus*; Lane 8: *L. plantarum*; Lane 9: negative control using nuclease-free water. The lane indicated by the red arrow represents *A. hydrophila*.

The genomic DNA of *A. hydrophila*, *A. veronii*, *V. harveyi*, *P. fluorescens*, *B. subtilis*, *B. cereus*, and *L. plantarum* was analyzed in the MIRA assay. The *A. hydrophila* and nuclease-free water were used as positive and negative controls, respectively. The *A. hydrophila* displayed distinct single bands in the agarose gel electrophoresis, whereas *A. veronii*, *V. harveyi*, *P. fluorescens*, *B. subtilis*, *B. cereus*, and *L. plantarum* did not display any bands, as illustrated in Figure 3, which suggested that the developed MIRA assay had high specificity.

#### 3.1.3. The Basic MIRA Assay with High Sensitivity

**Figure 4:**
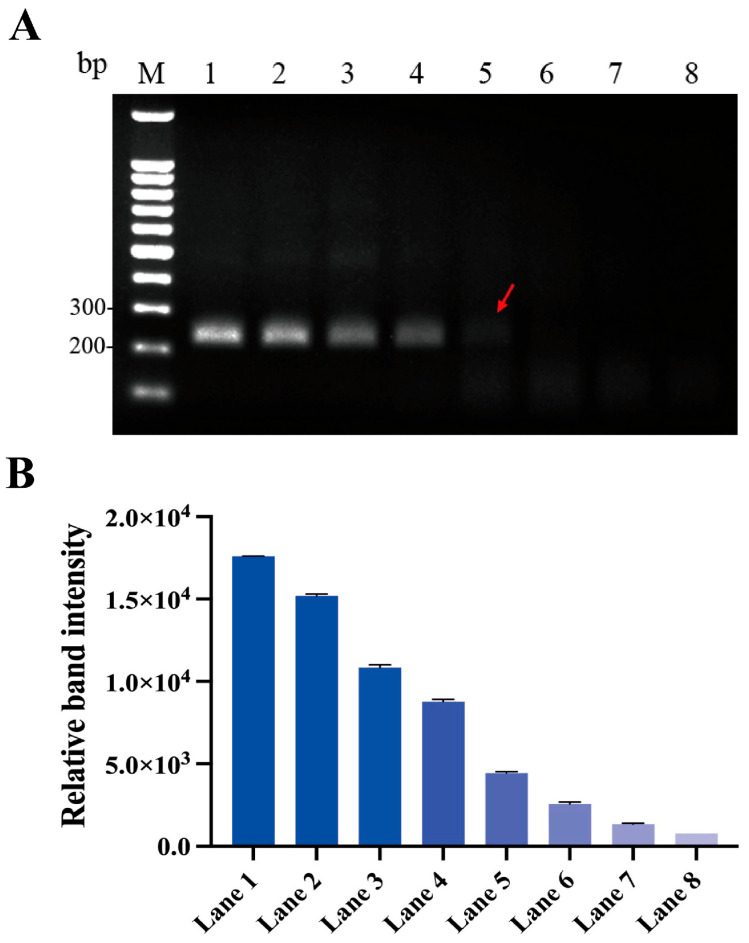
Sensitivity of the basic MIRA assay. (**A**) Agarose gel electrophoresis image; (**B**) Relative band intensity. M: 1000 bp DNA Marker; Lane 1: 10 pg/μL; Lane 2: 1 pg/μL; Lane 3: 100 fg/μL; Lane 4: 10 fg/μL; Lane 5: 1 fg/μL; Lane 6: 0.1 fg/μL; Lane 7: 0.01 fg/μL; Lane 8: Negative control using nuclease-free water. The lane indicated by the red arrow represents the detection limit concentration.

The plasmid pUC57-aerA, with concentrations ranging from 0.01 fg/μL to 10 pg/μL, was used to determine the detection limit of the basic MIRA assay. The amplification of the recombinant plasmid pUC57-aerA at concentrations of 10 pg/μL, 1 pg/μL, 100 fg/μL, 10 fg/μL, and 1 fg/μL using primer pair AH-aerA-F2/R2 all produced distinct single band with the size between 200 and 300 bp in the agarose gel electrophoresis. We observed a decrease in band brightness with a diminishing template concentration, indicating that the amplification efficiency of the basic MIRA for *A. hydrophila* is positively correlated with template concentration. The amplified product of the recombinant plasmid pUC57-aerA at a concentration of 1 fg/μL produced a very faint band with the expected size, suggesting that the detection limit of the basic MIRA assay for the recombinant plasmid pUC57-aerA is approximately 1 fg/μL. These results demonstrate that the developed MIRA assay has high sensitivity, making it suitable for the detection of *A. hydrophila*. These results demonstrate that the developed MIRA assay has high sensitivity, making it suitable for the detection of *A. hydrophila*, as shown in Figure 4.

### 3.2. Fluorescent MIRA Assay

#### 3.2.1. Optimal Primer Set for the Fluorescent MIRA Assay

**Figure 5:**
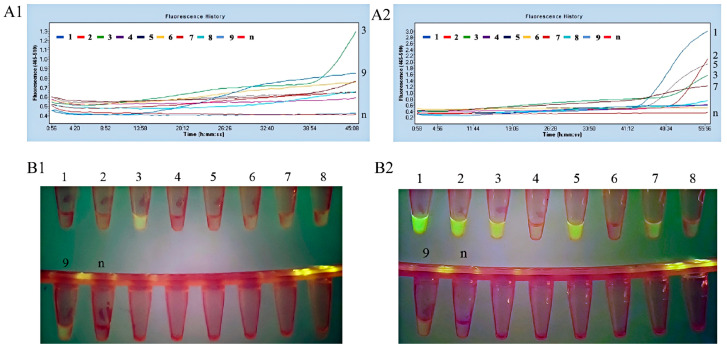
Primer screening for the fluorescent MIRA assay using *A. hydrophila* genomic DNA as the template. The amplification results of MIRA combined with fluorescence read-out using 50 ng/μL *A. hydrophila* genomic DNA as the template (**A1**). Direct visual observation under UV light of the amplification products of MIRA using 50 ng/μL *A. hydrophila* genomic DNA as the template (**B1**). The amplification results of MIRA combined with fluorescence read-out using 500 pg/μL *A. hydrophila* genomic DNA as the template (**A2**). Direct visual observation under UV light of the amplification products of MIRA using 500 pg/μL *A. hydrophila* genomic DNA as the template (**B2**). Primer pairs 1–9 correspond to the following combinations: (1) AH-aerA-F1/R1, (2) AH-aerA-F1/R3, (3) AH-aerA-F1/R2, (4) AH-aerA-F2/R1, (5) AH-aerA-F2/R3, (6) AH-aerA-F2/R2, (7) AH-aerA-F3/R1, (8) AH-aerA-F3/R3, (9) AH-aerA-F3/R2, (n) Negative control using nuclease-free water.

The three upstream primers and three downstream primers were used in different combinations with probe to amplify *A. hydrophila* DNA at concentration of 50 ng/μL (Figure 5(A1,B1)) and 500 pg/μL (Figure 5(A2,B2)) in the fluorescent MIRA assay. In the fluorescent MIRA detection of *A. hydrophila* genomic DNA at a concentration of 50 ng/μL, the amplification products of primer pairs 3 and 9 exhibited pronounced fluorescence. In the MIRA detection of *A. hydrophila* genomic DNA at a concentration of 500 pg/μL, the amplification products of primer pairs 1, 2, 3, 5, and 7 all exhibited pronounced fluorescence. In summary, primer pair 3 (AH-aerA-F1/R2) consistently demonstrated significant fluorescence in the fluorescent MIRA detection of *A. hydrophila* genomic DNA at low and high concentrations. Therefore, primer 3 (AH-aerA-F1/R2) was selected for further experiments.

#### 3.2.2. The Fluorescent MIRA Assay with High Specificity

**Figure 6:**
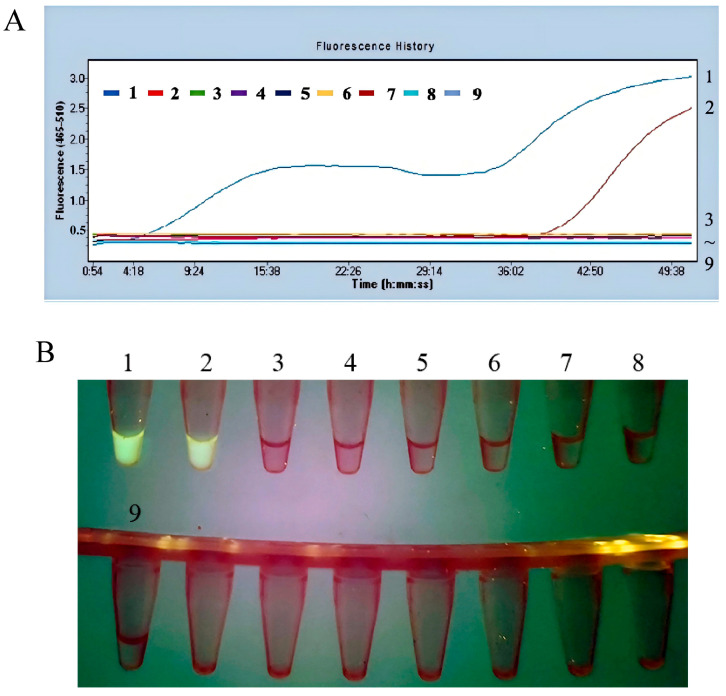
Specificity of the fluorescent MIRA assay. (**A**) The amplification results of fluorescent MIRA in fluorescence read-out. (**B**) Direct visual observation under UV light of the amplified products of fluorescent MIRA. 1: pUC57-aerA; 2: *A. hydrophila*; 3: *A. veronii*; 4: *V. harvey*; 5: *P. fluorescens*; 6: *B. subtilis*; 7: *B. cereus*; 8: *L. plantarum*; 9: negative control using nuclease-free water.

The specificity of the fluorescence MIRA method was validated using genomic DNA from *A. veronii*, *V. harveyi*, *P. fluorescens*, *B. subtilis*, *B. cereus*, and *L. plantarum*. The plasmid pUC57-aerA was served as the positive control, and nuclease-free water was used as the negative control. The results showed that both *A. hydrophila* and the positive control exhibited strong fluorescence, while no fluorescence was detected in the other bacterial samples and the negative control, as shown in Figure 6. These findings demonstrated that the fluorescence MIRA assay developed in this study possesses high specificity for the detection of *A. hydrophila*.

#### 3.2.3. The Fluorescent MIRA Assay with High Sensitivity

**Figure 7:**
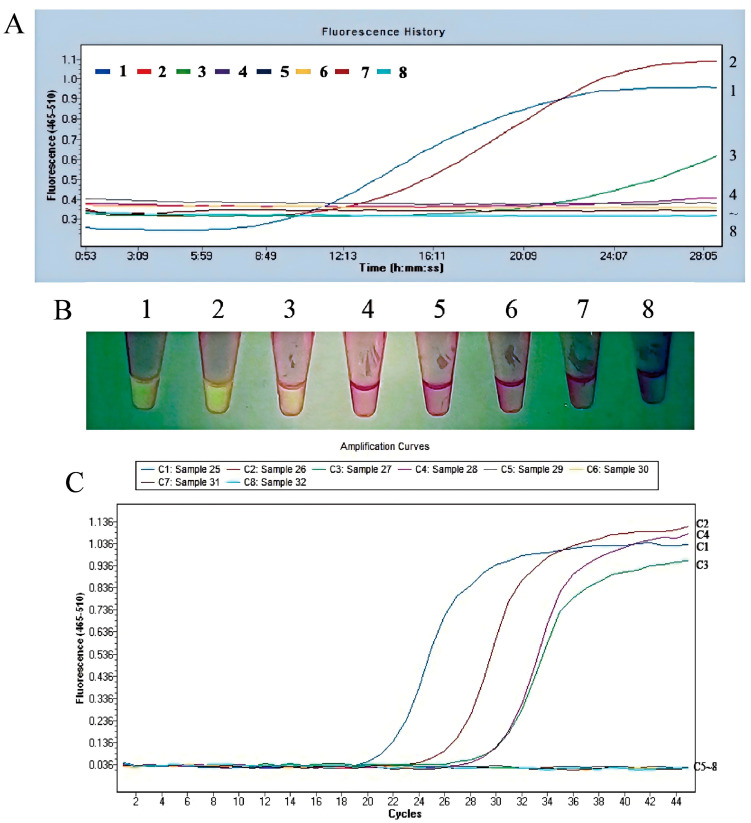
Sensitivity of the fluorescent MIRA assay. The 10-fold dilutions of plasmid pUC57-aerA, starting from a concentration of 100 fg/μL were used as the template for the fluorescent MIRA assay. (**A**) The amplification results of fluorescent MIRA in fluorescence read-out. 1: 100 fg/μL, 2: 10 fg/μL, 3: 1 fg/μL, 4: 1 × 10^−1^ fg/μL, 5: 1 × 10^−2^ fg/μL, 6: 1 × 10^−3^ fg/μL, 7: 1 × 10^−4^ fg/μL, 8: Negative control using nuclease-free water. (**B**) Direct visual observation under UV light of the amplified products of fluorescent MIRA. (**C**) Real-time PCR amplification curve. C1: 100 fg/μL, C2: 10 fg/μL, C3: 1 fg/μL, C4: 1 × 10^−1^ fg/μL, C5: 1 × 10^−2^ fg/μL, C6: 1 × 10^−3^ fg/μL, C7: 1 × 10^−4^ fg/μL, C8: Negative control using nuclease-free water.

The plasmid pUC57-aerA, with concentrations ranging from 100 fg/μL to 1 × 10^−4^ fg/μL, was used to evaluate the detection limit of the fluorescent MIRA assay and compared with the real-time PCR assay. The detection limit for *A. hydrophila* using real-time PCR was determined to be 0.1 fg/μL plasmid pUC57-aerA, as shown in Figure 7C. The amplification curve of fluorescent MIRA for the 1.0 × 10^−1^ fg/μL plasmid showed no detectable fluorescence signal (Figure 7A). These findings indicated that the detection limit of the fluorescent MIRA assay was 1 fg/μL, which was 10 times higher than that of real-time PCR. Consequently, the fluorescent MIRA assay developed in this study demonstrated high sensitivity and was suitable for the detection of *A. hydrophila*.

## 4. Discussion

This study established two MIRA-based detection methods for *A. hydrophila*, demonstrating high specificity and sensitivity through systematic validation. The basic MIRA assay utilized agarose gel electrophoresis for result visualization, retaining PCR-equivalent reliability while eliminating thermal cycling requirements to enhance operational efficiency. The fluorescent MIRA variant further accelerated detection through real-time signal monitoring, reducing interpretation time from several hours (nearly two hours) to within one hour via light-activated visualization.

The selection of the *aerA* gene as the detection target was based on its critical role in pathogenicity. It is a polypeptide with hemolytic, enterotoxic, and cytotoxic properties, considered an essential virulence factor capable of inducing host cell apoptosis and causing fish diseases [33,34]. Mutant strains of *A. hydrophila* deficient in the gene encoding *aerolysin* exhibited significantly reduced virulence compared to the wild-type strains in mice [35]. Our primer design targeting conserved *aerA* regions aligned with established detection strategies [36], including electrochemical biosensor approaches [10], ensuring specificity for pathogenic strains.

Various molecular diagnostic techniques for *A. hydrophila* are currently available, as summarized in Table 2. In this study, the fluorescent MIRA assay developed for the detection of *A. hydrophila* exhibited a detection limit 1 fg/μL for the recombinant plasmid pUC57-aerA, corresponding to 3.1 × 10^2^ copies/μL, demonstrating lower sensitivity compared to the real-time PCR, which had a detection limit of 0.1 fg/μL. However, the fluorescent MIRA assay required only 20 min, approximately one-third of the time needed for real-time PCR. These results suggest that fluorescent MIRA provides a more rapid and practical approach for the detection of *A. hydrophila*.

To date, this study has only evaluated detection performance using synthetic and purified DNA samples. The assay’s effectiveness in complex environmental samples—such as aquaculture water—remains unverified. Specificity was tested against a limited set of waterborne bacteria, yet natural environments contain diverse microorganisms that may cause interference. Future work will expand the validation of primer set to include a broader range of bacterial species and real environmental samples to better assess specificity, reliability, and practicality. Further optimizations will be made to enhance accuracy and robustness for real-world applications.

Aerolysin, a water-soluble pore-forming toxin secreted by *A. hydrophila*, plays a critical role in the pathogenesis of infections caused by this bacterium and is implicated in the onset of various diseases in host organisms [44]. Studies have demonstrated that the *aerA* gene, which encodes *aerA*, exhibits a high degree of specificity among common aquatic pathogens. Notably, when the *aerA* gene is deleted, the resulting mutant strains show significantly reduced pathogenicity compared to their wild-type counterparts, underscoring the essential function of this toxin in virulence [45]. Furthermore, a study conducted by Singh et al. in 2008 [36] revealed that *aerA* was detected in all *A. hydrophila* strains isolated from fish exhibiting hemorrhagic septicemia and in 60% of strains obtained from healthy fish. These findings strongly support a positive correlation between the presence of *aerA* and the pathogenic potential of *A. hydrophila* [36]. Non-pathogenic *A. hydrophila* can also encode the *aerA* gene, making it possible for false positives to occur when predicting disease risk solely based on the presence or absence of MIRA signals. Therefore, the establishment of control groups is necessary. In practical testing (e.g., colloidal gold), negative controls under normal farming conditions (qualified water or healthy fish) and positive controls under disease outbreak conditions (contaminated water or infected fish) should serve as the criteria for interpreting actual detection results, rather than relying on the presence or absence of MIRA signals. Therefore, our subsequent work will focus on actual aquaculture practices, aiming to determine the detection limit of the *aerA* gene under both normal and disease-onset conditions. This will allow us to further refine the MIRA-based *aerA* detection technology for improved disease monitoring. Ultimately, such advancements aim to enhance the predictive capability for disease outbreaks and support more effective management strategies in aquaculture and other relevant settings.

## 5. Conclusions

In conclusion, the 20 min MIRA assays developed here provide a balanced combination of specificity (through *aerA* targeting), practicality (via isothermal operation), and adaptability (with dual detection formats). This technological advancement addresses pressing needs for point-of-care diagnostics in aquaculture disease management, offering substantial potential for early outbreak containment and preventive surveillance.

## Figures and Tables

**Table 1:** Primers and probe sequences of *A. hydrophila* used in this study.

Prime	Sequence (from 5′ to 3′)
AH-aerA-F1	TGCGGCCAACCAGTCATGGGCATCCCAGAACG
AH-aerA-F2	TACCACCACCTCCCTGTCGCAATCCGTGCGG
AH-aerA-F3	TGTCGCAATCCGTGCGGCCGACGGTGCCGG
AH-aerA-R1	CAACGCAGGAAGCCACTCAGGGTCAGGTCA
AH-aerA-R2	TTGTCCTTGTACGGCCCGATGACGAAGGTG
AH-aerA-R3	GGGCGATTGTCCGGATGGGTATACCAGGCAT
AH-aerA-F	GAGAAGGTGACCACCAAGAACA
AH-aerA-R	AACTGACATCGGCCTTGAACTC
AH-aerA-P	5′-CGGTGAAGATCGAGCTCTACAAGGCTGATA[FAM-dT][THF][BHQ-dT]CCTATCCCTATGAGT-3′C3spacer

**Table 2:** Comparison of methods for the detection of *A. hydrophila* by molecular detection techniques.

Detection Methods	Target Gene for Detection	Reaction Time	Sensitivity
PCR [37]	*aerA*	1 h	Genomic DNA concentration 100 fg/μL
Multiplex-PCR [38]	*aerA*	1.5 h	Genomic DNA concentration 8 × 10^2^ fg/μL
Quadruple PCR [39]	*ahpA*; *aerA*; *hlyA*; *16S rRNA*	2.3 h	Genomic DNA content 100 fg
Real-time fluorescence quantitative PCR [40]	*hlyA*	30 min	Bacterial quantification linear range 5.4 × 10^3^~5.4 × 10^8^ CFU/mL
Dual fluorescence quantitative PCR [41]	*16S rDNA*; *aerA*	1.2 h	Plasmid 10 copies/reaction
Loop-mediated isothermal amplification (LAMP) [42]	*aerA*	40 min	Genomic DNA concentration 0.559 ng/μL
Real-time recombinase polymerase amplification (real-time RPA) [43]	*haemolysin*	20 min	10^2^ copies of the *A. hydrophila* per reaction

## Data Availability

The original contributions presented in the study are included in the article, further inquiries can be directed to the corresponding authors.
